# Does body shadow improve the efficacy of virtual reality-based training with BTS NIRVANA? A pilot study

**DOI:** 10.1097/MD.0000000000008096

**Published:** 2017-09-22

**Authors:** Margherita Russo, Rosaria De Luca, Antonino Naro, Francesca Sciarrone, Bianca Aragona, Giuseppe Silvestri, Alfredo Manuli, Alessia Bramanti, Carmela Casella, Placido Bramanti, Rocco Salvatore Calabrò

**Affiliations:** a IRCCS Centro Neurolesi Bonino Pulejo; b Stroke Unit, Policlinico Universitario, Messina, Italy.

**Keywords:** body shadow, cognitive, rehabilitation, stroke, virtual reality

## Abstract

**Background:**

Aim of the present study was to evaluate whether the presence of body shadows during virtual reality (VR) training with BTS NIRVANA (BTs-N) may lead to a better functional recovery.

**Methods:**

We enrolled 20 poststroke rehabilitation inpatients, who underwent a neurocognitive-rehabilitative training consisting of 24 sessions (3 times a week for 8 weeks) of BTs-N. All the patients were randomized into 2 groups: semi-immersive virtual training with (S-IVT_S_ group) or without (S-IVT group) body shadows. Each participant was evaluated before (T0) and immediately (T1) after the end of the training (Trial Registration Number: NCT03095560).

**Results:**

The S-IVT_S_ group showed a greater improvement in visuo-constructive skills and sustained attention, as compared with the S-IVT group. The other measures showed nonsignificant within-group and between-group differences.

**Conclusion:**

Our results showed that body shadow may represent a high-priority class of stimuli that act by “pushing” attention toward the body itself. Further studies are needed to clarify the role of body shadow in promoting the internal representation construction and thus self-recognition.

## 1 Introduction

Stroke is one of the leading causes of death and disability, and this has been described as a worldwide epidemic.^[1]^ The effects of a stroke may include sensory, motor, and cognitive impairment, as well as a reduced ability to perform self-care and participate in social and community activities.^[2]^ Although most recovery is thought to occur in the first few weeks after stroke, patients may make improvements on functional tasks many months after having a stroke,^[3]^ given that neuroplasticity exists also in the chronic phase.

Cognitive impairment and neuropsychiatric symptoms, including attention and concentration, memory, spatial awareness, perception, praxis, and executive functioning deficits, frequently occur in poststroke patients. Indeed, about two-thirds of stroke survivors may experience cognitive changes.^[4,5]^ These changes have a significant impact on long-term outcome and appear to be associated with an increased risk of disability, depression, and quality of life.^[6]^ Early recognition and monitoring of cognitive changes in stroke is necessary to optimize patient care, and this can guide therapy and rehabilitation strategies.^[7]^

The use of alternative rehabilitation tools is a growing field, given that the standard rehabilitation for stroke is not always so effective in improving functional outcomes.^[8]^ Virtual reality (VR) has a prominent role in promoting functional recovery after stroke, as it can integrate multisensory stimulation to provide a realistic environment and to enrich motivational training improving patients’ adherence to the rehabilitative program.^[8]^

VR has been defined as the “use of interactive simulations created with computer hardware and software to present users with opportunities to engage in environments that appear and feel similar to real-world objects and events,”^[9]^ and it is believed to optimize motor learning.^[10]^ VR offers clinicians the ability to control and grade tasks to challenge the user, as VR programs often incorporate multimodal feedback provided in real time.

However, there are few studies on the role of VR training in cognitive rehabilitation (CR) of stroke patients, though an improvement in balance and gait has been reported.^[11]^ Virtual rehabilitation system provides 3 different types of information to the patient: movement visualization (MV), performance feedback, and context information.^[12]^ Movement observation plays an important role for central sensory stimulation therapies, such as mirror therapy or mental training; indeed, the observation or imagination of body movements facilitates motor recovery^[13–15]^ and provides new possibilities for cortical reorganization. Thus, it appears that MV is a key element of VR-based rehabilitation intervention.^[11]^ The presence of body shadow, considered as MV, has recently been a target of an increasing number of studies, mainly showing that information conveyed by shadows can support several tasks performed in everyday life.^[16]^ When applied to body shadows, the shadow-correspondence problem may thus be central to a perceptual decision that promotes self-identification and self-recognition.^[16]^

The aim of the present study was to detect the differences in functional outcomes between the presence or not of body shadow during a VR training with BTS NIRVANA (BTs-N), using an interactive-semi-immersive program.

## 2 Materials and methods

We enrolled 20 poststroke inpatients (12 males and 8 females, mean age 64 ± 6), who attended our Robotic and Behavioral Neurorehabilitation Center from June 2016 to January 2017. The inclusion criteria were ischemic or hemorrhagic stroke in the chronic phase (at least 6 months after the ischemic/hemorrhagic accident) with a severe upper limb impairment; presence of moderate-to-mild cognitive impairment (Mini-Mental State Examination [MMSE] from 11 to 26) assessed after the stroke; and absence of disabling sensory alterations (ie, audio-visual deficits) and severe psychiatric illness.

The exclusion criteria were age >75 years, presence of severe medical and psychiatric illness, and intake of drugs potentially affecting neurocognitive functions. The study was approved by the Local Ethical Committee, and registered on the www.clinicaltrials.com (NCT03095560). All the patients provided informed consent to enter the study. All the participants were randomized (by using a randomized block design) into 2 groups: semi-immersive virtual training with (S-IVT_S_) or without (S-IVT) body shadow, and underwent the same conventional physiotherapy program in addition to a VR neurocognitive-rehabilitative training (24 sessions of BTs-N, 45 minutes daily, 3 times a week, for 8 weeks). Each participant was evaluated by skilled neuropsychologist and neurologist before (T0) and immediately (T1) after the end of the training. Primary outcomes were assessed using the Montreal Cognitive Assessment Test (MoCA) and the Functional Independence Measure (FIM). In addition, we administered the Frontal Assessment Battery (FAB) to assess executive functions, attentive matrices (AM), and Trail Making Test (TMTA, TMTB, and TMTB-A) to measure the attention process, and the Trunk Control Test (TCT) and Motricity Index (MI) for upper and lower limbs to evaluate motor function.

NIRVANA is a device based on optoelectronic infrared sensors, through which the patient can interact with a virtual scenario. The system is connected to a projector or a big screen, reproducing an interactive series of exercises, thanks to an infrared video camera analyzing the patient's movements, it creates interactivity. In the BTs-N device we used, the projector was located behind the patient in the S-IVT_S_ group, projecting the shadow of the patient on the screen, whereas the projector was located in front of the patient in the S-IVT group, being thus the shadow not visible. The virtual cognitive rehabilitative program with BTs-N we carried out in both the groups is described in details in Table 1.

**Table 1:**
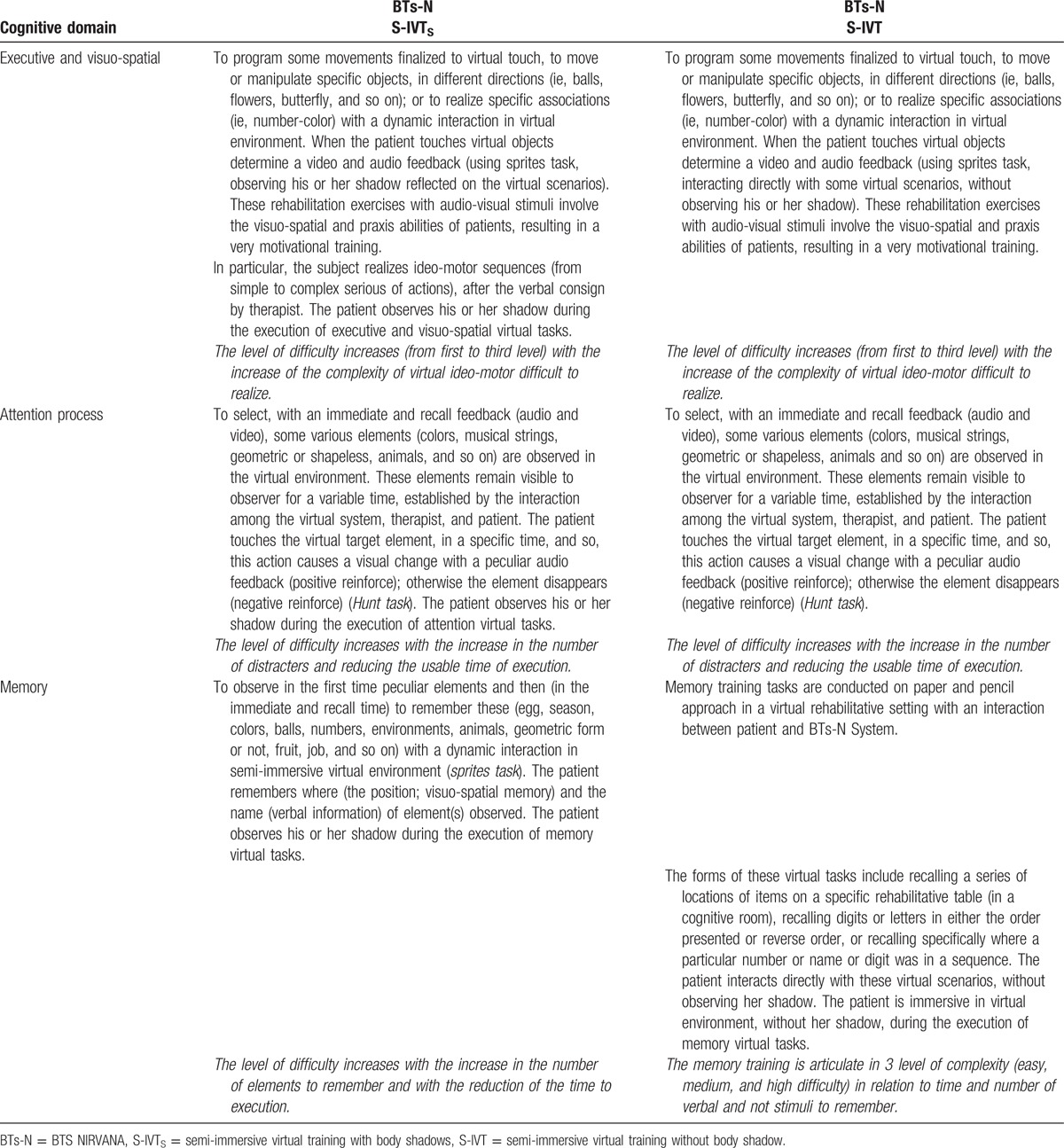
Virtual Cognitive Rehabilitative Program used in patients undergoing BTs-N System.

A Wilcoxon signed rank (WSR) test was employed to compare within-group changes from baseline (T0) to post-treatment (T1). Baseline between-group differences were tested through the Mann-Whitney *U* test (MWU). The MWU was also used for between-group comparisons by computing the differences between T1 and T0 for all outcome measures. We set the alpha-level for significance at .05, adjusted for multiple comparisons using a Bonferroni correction. Data are reported as median and interquartile distribution.

To understand how large the differences between S-IVT_S_ and S-IVT were, we calculated the effect size Cohen *d*. Given the relatively small sample size, we applied Hedge *g* correction to the biased effect size estimate. Effect size is simply a way of quantifying the size of the difference between 2 groups, that is, it is a quantitative measure of the strength of a phenomenon. Indeed, the amount of variation found within- or between-group is quantified in the calculation of the effect size. For these reasons, effect size is an important tool in reporting and interpreting effectiveness of a treatment. Specifically, Cohen *d* is defined as the difference between 2 means divided by a standard deviation (SD) for the data.

According to the available literature on the effects of VR training in patients with stroke, we calculated a minimal sample size based on the asymptotic relative efficiency (ARE) of the MWU relative to the *t* tests. We estimated that a minimal sample of 20 participants per group would be necessary to confirm the data of our pilot study.

## 3 Results

Clinical data at T0 and T1 are summarized in Table 2. There was a large effect size for the improvement in MoCA in the S-IVT_S_ group (*P* < .001, *d* = 0.9) at T1 as compared with T0, which was more evident than that observed in S-IVT group at the same interval (*P* = .02, *d* = 0.6; MWU: *P* = .03, *d* = 0.6). In particular, only S-IVT_S_ group showed a large effect size for the amelioration in visuo constructive (*P* = .002, *d* = 0.7; MWU: *P* = .02, *d* = 0.9), and a moderate effect size for the improvement in attention (*P* = .001, *d* = 0.6; MWU: *P* = .02, *d* = 0.6). FIM slightly improved in both the groups, without reaching the statistical significance.

**Table 2:**
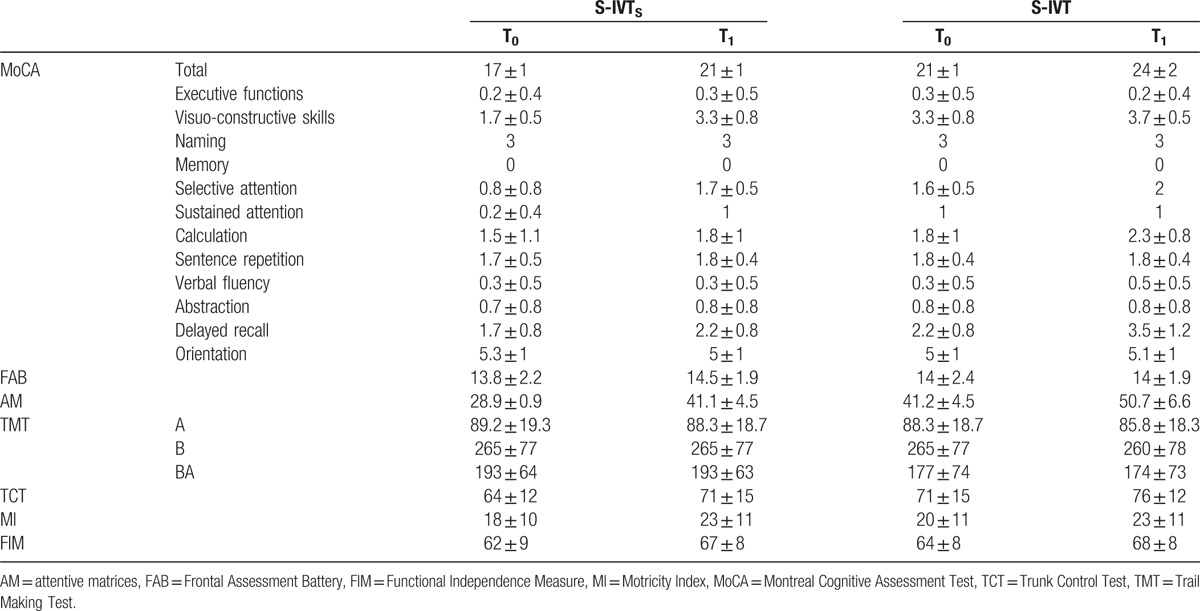
Clinical data of the experimental and control group at T0 and T1.

Both the groups improved in all secondary outcome measures at T1 as compared with T0, albeit only AM (S-IVT_S_: *P* < .001, *d* = 0.8; S-IVT: *P* = .02, *d* = 0.6) and MI (S-IVT_S_: *P* < .001, *d* = 0.6; S-IVT: *P* = .002, *d* = 0.4) reached the statistical significance with moderate-to-large effect size. Such improvements were more evident in the S-IVT_S_ than S-IVT group (MWU for AM: *P* = .02, *d* = 0.6; MWU for MI: *P* < .001, *d* = 0.8).

## 4 Discussion

Our results suggest that the use of VR training with BTs-N is useful in improving cognitive abilities (specifically, visuo-constructive abilities and attention) in the poststroke rehabilitation, as suggested by the moderate-to-large effect size in nearly all outcome measures (favoring S-IVT_S_).

Despite more than 40% of stroke survivors may experience a decline in cognitive function, the majority of the published data have paid attention mainly to motor recovery.^[17]^ Cognition is not a unitary concept, but it incorporates multiple domains, including attention, executive functions, problem solving, visual-spatial ability, language, social cognition, and emotions.^[18]^

Executive function is the term generally used to describe the brain processes that we employ to organize ourselves and solve problems. Moreover, executive function is strictly related to other important “subfunctions,” including inhibition (ie, the ability to suspend prepotent/default responses), mental flexibility (ie, the ability to switch back and forth between rules or response sets), and working memory. These functions are critically important for quality of life, as they are implicated in job performance, social relationships, and both basic and instrumental activities of daily living.^[19]^

Attention is the behavioral and cognitive process of selectively concentrating on a discrete aspect of information, whether deemed subjective or objective, while ignoring other perceivable information. Focalization and concentration of consciousness are of its essence.

Visuo-spatial ability is the capacity to understand, reason, and remember the spatial relations among objects or space that includes your own body parts.^[20–22]^

Besides the aforementioned cognitive alterations, memory deficits can also be considered a relatively common consequence of stroke,^[23]^ such problems being persistent, debilitating, and often difficult to treat.^[17]^ Although we used MMSE for the patients’ screening, we preferred MoCA for the assessment, given that such test is divided into subitems better investigating those domains we believed may benefit from BTs-N training, also taking into account that several studies have considered MoCA as a valuable tool for cognitive assessment in poststroke patients.^[24–26]^

CR therapy encompasses any intervention targeting the restoration, remediation, and adaptation of cognitive functions.

CR techniques may be classified in 2 main categories, that is, conventional/classic (paper/pencil exercises) and computer-assisted/not conventional (ie, computerized cognitive rehabilitation [CCR]), both based on the use of cognitive strategies to retrain or alleviate the patient's deficits in attention and concentration, visual processing, language, memory, reasoning and problem solving, and executive functions. Conventional techniques consist of manual exercises with the therapist, whereas computerized exercises train cognitive skills in gamelike programs. Computer use in CR extends to memory training, attention, problem solving, and job simulation. CCR uses multimedia and informatics resources with particular hardware systems and software, that is, ad hoc programs built to reactivate compromised neuropsychological functions.^[27]^

VR and interactive technologies have emerged as a valuable tool in stroke rehabilitation by providing the opportunity to practice cognitive and motor activities that cannot be practiced within the clinical environment, such as performing simulations of real-life scenarios and activities in urban virtual environments.^[28]^

Despite the growing scientific and engineering activity in VR-based systems, the majority of the studies were designed to address motor impairments.^[28,29]^ Indeed, according to a recent Cochrane review,^[8]^ there are only few randomized controlled studies that include CR and/or cognition assessment,^[30]^ though a positive effect of VR in poststroke balance and gait deficit has been reported.^[31,32]^

Timing of CR therapy is also noteworthy, as previous literature has indicated that functional recovery typically occurs in ∼95% of stroke patients within the first ∼3 months from the acute event.^[33]^

In fact, it is thought that most of the neurological recovery that occurs following a stroke is a direct result of brain plasticity and its ability to repair and reorganize,^[34]^ largely depending on the lesion severity and site.^[35]^

On the assumption that reorganization would occur to replace functions of a damaged brain part, a VR rehabilitation training may sustain the improvement in cognitive abilities through complex mechanisms.^[8]^ Action observation, in association with physical training, can enhance the effects of rehabilitation training after stroke^[36,37]^ and, more than just playing or training, the implementation of VR training may gear use-dependent neuroplasticity changes.^[8]^

In keeping with this argument, growing evidence suggests the role of “mirror neurons” during the execution or observation of actions performed by other individuals, thus enhancing motor and cognitive recovery.^[38]^

Thus, the more significant improvement in visuo-constructive abilities, attentive functions, and upper limb motricity in the S-IVT_S_ patients could be due to the shadow effect (ie, the patient's shadow on the screen while performing VR training) of BTs-N.

Body shadow processing can be reflected at the level of the human mirror neuron system, even when shadows are not relevant for the specific task.^[16]^ This issue makes body shadow potentially capable of contributing to the construction of the internal representation of body shape and its extension in space. When applied to body shadows, the shadow-correspondence problem may thus be central to a perceptual decision that promotes self-identification and self-recognition,^[39,40]^ potentially leading to better functional outcomes.

It is worth noting that not only diagnostic resources and treatments evolve, but also the rehabilitation techniques with therapies and devices, which in the future (and present) can help stroke patients, as demonstrated by our promising tool.

There are some limitations to acknowledge. The sample size is obviously small, as this is a pilot study. Therefore, larger sample trials should be fostered to confirm these interesting data. Baseline data were slightly different between the groups. This might obviously influence S-IVT aftereffects. However, baseline between-group differences were tested through the MWU, which did not disclose significant differences between the 2 groups. This fact may depend on either the relatively high SD or the small sample enrolled (as a pilot study). This aspect needs verification in future larger sample studies.

In conclusion, our results suggest that body shadow may represent a high-priority class of stimuli that act by “pushing” attention toward the body itself, thus contributing to cognitive and motor recovery. Further studies should be developed to clarify the role of body shadow in the self-recognition and the internal representation construction, which, in turn, can be considered future target of neurorehabilitation.
